# Genome architecture of *Lactobacillus plantarum* PS128, a probiotic strain with potential immunomodulatory activity

**DOI:** 10.1186/s13099-015-0068-y

**Published:** 2015-08-15

**Authors:** Wei-Hsien Liu, Chih-Hsien Yang, Ching-Ting Lin, Shiao-Wen Li, Wei-Shen Cheng, Yi-Ping Jiang, Chien-Chen Wu, Chuan-Hsiung Chang, Ying-Chieh Tsai

**Affiliations:** Institute of Biochemistry and Molecular Biology, National Yang-Ming University, No. 155, Sec 2, Linong Street, Taipei, 11221 Taiwan; Institute of Biomedical Informatics, National Yang-Ming University, No. 155, Sec 2, Linong Street, Taipei, 11221 Taiwan; Center for Systems and Synthetic Biology, National Yang-Ming University, Taipei, 11221 Taiwan; School of Chinese Medicine, China Medical University, Taichung, 40402 Taiwan; Probiotics Research Center, National Yang-Ming University, Taipei, 11221 Taiwan

**Keywords:** *Lactobacillus plantarum*, Probiotics, Immunomodulation, PS128, Draft genome sequence

## Abstract

**Background:**

Clinical and preclinical observations indicate that *Lactobacillus plantarum* has anti-inflammatory activity and may regulate the immune responses of its hosts when ingested. Recently, modification of teichoic acids (TAs) produced by *L. plantarum* was reported as a key to regulating the systemic immune response in mice. However, data linking TA-related genetic determinants and the immunomodulatory effect are limited. To provide genomic information for elucidating the underlying mechanism of immunomodulation by *L. plantarum*, we sequenced the genome of *L. plantarum* strain PS128.

**Results:**

The PS128 genome contains 11 contigs (3,325,806 bp; 44.42% GC content) after hybrid assembly of sequences derived with Illumina MiSeq and PacBio RSII systems. The most abundant functions of the protein-coding genes are carbohydrate, amino acid, and protein metabolism. The 16S rDNA sequences of PS128 are closest to the sequences of *L. plantarum* WCFS1 and B21; these three strains form a distinct clade based on 16S rDNA sequences. PS128 shares core genes encoding the metabolism, transport, and modification of TAs with other sequenced *L. plantarum* strains. Compared with the TA-related genes of other completely sequenced *L. plantarum* strains, the PS128 contains more lipoteichoic acid exporter genes.

**Conclusions:**

We determined the draft genome sequence of PS128 and compared its TA-related genes with those of other *L. plantarum* strains. Shared genomic features with respect to TA-related subsystems may be important clues to the mechanism by which *L. plantarum* regulates its host immune responses, but unique TA-related genetic determinants should be further investigated to elucidate strain-specific immunomodulatory effects.

**Electronic supplementary material:**

The online version of this article (doi:10.1186/s13099-015-0068-y) contains supplementary material, which is available to authorized users.

## Background

*Lactobacillus plantarum*—a rod-shaped, facultative heterofermentative and anaerobic, Gram-positive lactic acid bacterium—is found in a range of environmental niches including gastrointestinal tracts, as well as in various food products. It is generally recognized as safe on the basis of the long history of human consumption of *Lactobacilli* in food. In addition to being safe, *L. plantarum* is easy to be cultured and is therefore of great interest to researchers in the food industry [1]. Furthermore, the *L. plantarum* genome is relatively large compared with the genomes of other *Lactobacilli*, and this species may exhibit complicated regulatory responses to environmental changes [2], which may also impact microbe–host interactions.

*Lactobacillus plantarum* has been shown to inhibit inflammation [3–5]. For example, *L. plantarum* 299v eases abdominal pains in patients with irritable bowel syndrome, an illness characterized by overactive inflammatory responses in the gut [6]. In addition, live and heat-killed *L. plantarum* MYL26, as well as bacterial cell wall extracts, increase the tolerance of human epithelial cells to endotoxin-induced inflammation by regulating gene expression in the cells, suggesting that entities on the bacterial cell wall contribute to the anti-inflammatory activity of the bacteria [7]. Interestingly, we have found that *L. plantarum* PS128—which was isolated from *fu*-*tsai*, a traditional Taiwanese fermented vegetable food product—reduces lipopolysaccharide-induced pro-inflammatory cytokine production in an RAW 264.7 mouse macrophage cell model (Additional file 1). It is notable that the teichoic acids (TAs) produced by *L. plantarum* are implicated in the activation of immune signaling [8]; and a recent study in mice shows that defective d-alanylation of TAs abolishes both pro-and anti-inflammatory responses of the host immune system [9], suggesting that the processes including modification in the TAs biosynthesis are involved in the immunomodulatory effects of *L. plantarum*.

Teichoic acids are the major class of cell surface glycopolymers of Gram-positive bacteria that comprised of two types: the wall-TAs (WTAs) and the lipo-TAs (LTAs) [10, 11]. The backbones of WTAs are initially synthesized by proteins encoded by *tar* or *tag* homologues, whereas those of LTAs are synthesized by *ltaS*. Following the modification or transport by respective proteins in different TAs types, the WTAs and the LTAs are eventually anchored to the peptidoglycan layer and the cytoplasmic membrane, respectively [8, 11, 12] (Additional file 2). However, the degree to which the TA-related gene products of *L. plantarum* contribute to the immunomodulatory activity remains to be determined (Additional file 2).

Herein, we report the draft sequence of the *L. plantarum* PS128 genome and a comparison of its TA-related genes with those of other *L. plantarum* strains. The goal of the study is to identify possible genetic determinants involved in immunomodulation of *L. plantarum*.

## Methods

### Sample collection and isolation of PS128

*Lactobacillus plantarum* PS128 was isolated from *fu*-*tsai* [13]. This traditional fermented food is produced in sealed jars filled with sun-dried mustard plants and salt, which are stored upside down for at least 3 months. After this fermentation period, lactic acid bacteria were isolated by homogenization of the fermented mixtures. The homogenized samples were serially diluted and plated out on sterile de Man Rogosa Sharpe agar (Becton, Dickinson and Company, Sparks, MD, USA). Colonies on the plates were subcultured at least three times for purification. Then single colonies were inoculated into de Man Rogosa Sharpe broth for further use.

### Identification of PS128

Microscopy revealed one of the purified colonies to contain rod-shaped bacteria and analysis of its fermentation profile with an API 50 CHL kit (bioMerieux, Lyon, France) allowed us to classify it as *L. plantarum* subsp. *plantarum*. To sequence the16S rDNA, we cultured PS128 aerobically at 37°C for 16 h before DNA extraction. Genomic DNA was extracted by means of a phenol/chloroform extraction method, as described previously [13]. The genomic DNA pellet was resuspended in TE buffer (10 mM Tris–HCl containing 1 mM EDTA, pH 8.0) and stored at −20°C until use.

The 16S rDNA sequence was amplified by means of polymerase chain reaction with primers 8F (5′-AGAGTTTGATCMTGGCTCAG-3′) and 15R (5′-AAGGAGGTGATCCARCCGCA-3′) [13] under the following conditions: denaturation at 95°C for 10 min, followed by 35 cycles at 95°C for 1 min, 50°C for 1 min, and 72°C for 1.5 min, with a final 3 min of extension at 72°C. The amplified product was purified and sequenced on an ABI 3730XL capillary DNA sequencer at the Sequencing Core Facility of National Yang-Ming University Genome Center. PS128 was confirmed to be a *L. plantarum* species by searching its 16S rDNA sequence with the Basic Local Alignment Search Tool (BLAST) web service of the National Center for Biotechnology Information (NCBI). Live PS128 was deposited according to the requirements of the Budapest Treaty at the Leibniz Institute DSMZ—German Collection of Microorganisms and Cell Cultures (Braunschweig, Germany; accession number DSM 28632).

### Genome sequencing

Purity of genomic PS128 DNA was assessed in terms of the A260/A280 ratio with a NanoDrop 1000 spectrophotometer (Thermo Fisher Scientific Inc.). The extracted genomic DNA was sequenced with both a MiSeq system (Illumina, Inc.) and an RSII system (Pacific Biosciences of California, Inc.) to generate 4,004,838 short reads (251 nt on average) in pairs and 163,478 PacBio reads (5,668 nt on average).

### Genome assembly and annotation

Before genome assembly, we trimmed the Illumina pair-end reads and the PacBio reads by setting the maximal error probability as 0.05 (equivalent to a Phred quality score of 13) and allowing at most two ambiguous nucleotides in CLC Genomics Workbench (ver. 7.0.3, Qiagen). First, the trimmed Illumina reads were de novo assembled (with a word size of 22 nt and a bubble size of 250 nt) into 35 contigs (with lengths of >1,000 bp and with >200-fold coverage). We manually classified the 35 contigs as 24 chromosome-derived contigs and 11 plasmid-like contigs based on BLAST search results against the non-redundant nucleotide database at NCBI. Using the Join Contigs function in the CLC Genome Finishing Module (Qiagen) with PacBio reads for guidance and a BLAST word size of 50 nt, the resulting 24 chromosome-derived contigs were further merged into 2 chromosome-derived super-contigs of 2,312,345 and 886,453 bp, respectively. The remaining 11 plasmid-like contigs were assembled into 9 plasmid-derived contigs. The resulting PS128 draft genome was uploaded to NCBI GenBank and annotated by means of the NCBI Prokaryotic Genome Annotation Pipeline (PGAP) [14] and the Rapid Annotation using Subsystem Technology (RAST) service [15] (with the classic annotation scheme, ClassicRAST). The functional categories of protein-coding genes were assigned by homology searching against the NCBI Clusters of Orthologous Groups (COG) database [16] and the SEED database [17]. TA-related genes were defined as they were annotated in the complete chromosome sequences of *L. plantarum* strains (16, B21, JDM1, P-8, ST-III, WCFS1, and ZJ316) and in the annotations of PS128 from the NCBI PGAP and the SEED RAST service. These annotations were further confirmed by BLAST analysis.

### Phylogenetic analysis based on 16S rDNA sequences

We collected full 16S rDNA sequences from *Lactobacilli* complete genomes (7 *Lactobacillus plantarum*, 9 *Lactobacillus casei*, 4 *Lactobacillus acidophilus*, and 3 *Lactobacillus salivarius*). Because most species of *Lactobacilli* have more than one copy of 16S rDNA in their genomes, we used multiple sequence alignments of all the 16S rDNA sequences for each species to obtain representative consensus sequences (using the cons application in the EMBOSS package [18]) before phylogenetic analysis. If a species had more than one representative sequence in the alignment of the 16S rDNA sequences, we labeled the 16S rDNA sequence patterns as type I, II, and so on. Eventually, we obtained 33 distinct 16S rDNA sequences. The evolutionary analyses of 16S rDNA sequences were carried out in MEGA6 [19]. The neighbor-joining method was used to infer the evolutionary history. We performed 1,000 replicates of the bootstrap test to obtain the percentage of replicates in which the associated taxa were clustered together. The evolutionary distances were computed by means of the maximum composite likelihood method and are in units of numbers of base substitutions per site.

### Quality assurance

Before whole-genome sequencing (Illumina MiSeq and PacBio RSII), the result of Sanger sequencing of PS128 16S rDNA was confirmed to be more than 99% similar to previously submitted sequences of *L. plantarum* when searching against the NCBI nonredundant nucleotide database.

### Initial findings

#### General features of PS128 genome

**Fig. 1:**
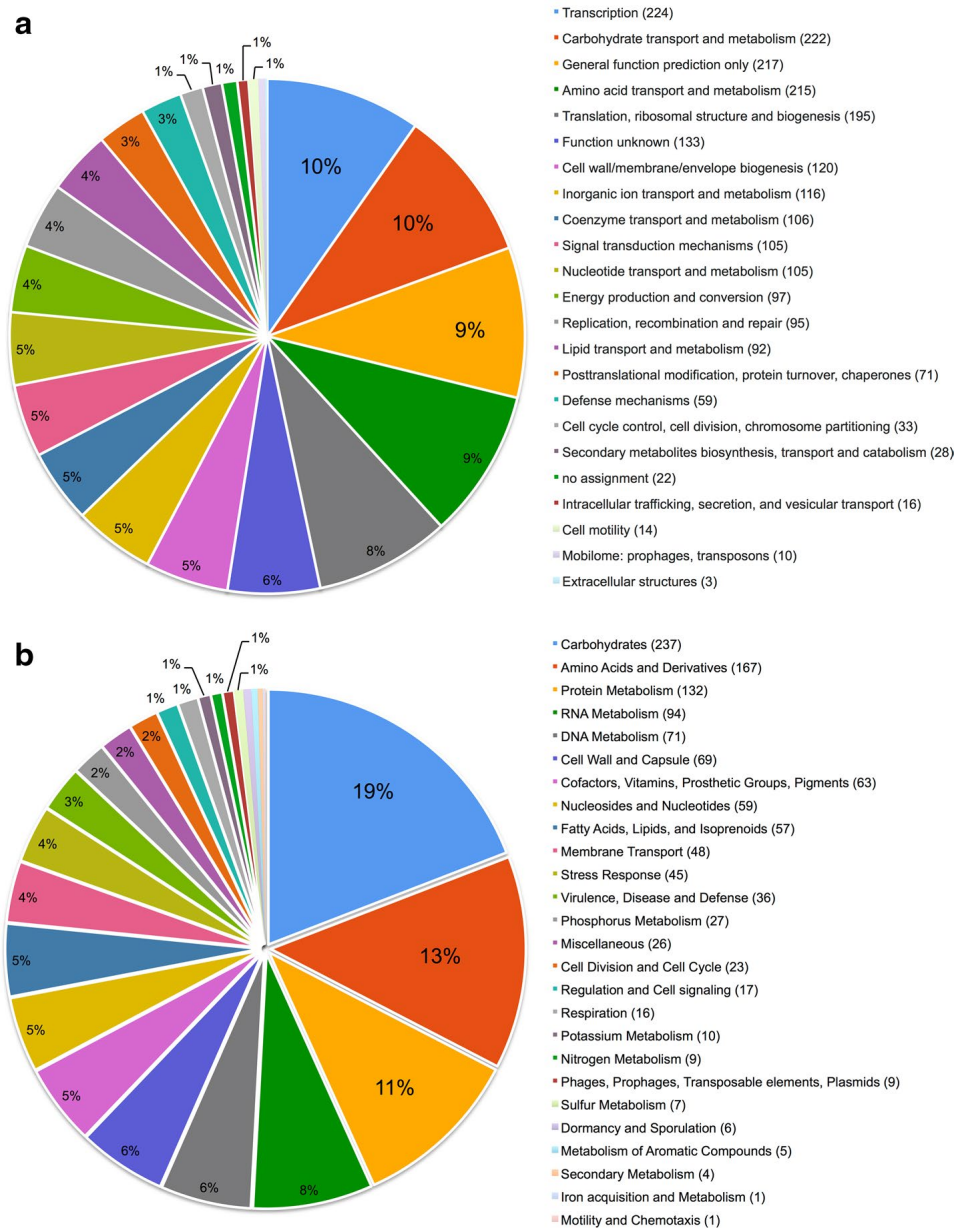
Functional distribution of the PS128 coding genes based on (**a**) COG functional categories and (**b**) SEED subsystems. There were 77.48 and 39.75% protein-coding genes assigned with COG and SEED functional categories, respectively.

The genome sequence reads of *L. plantarum* PS128 were assembled into two chromosomal contigs and nine contigs for potential plasmids after hybrid assembly of reads generated by means of Illumina MiSeq (4,004,838 reads) and PacBio RSII (163,478 reads). The chromosome size of PS128 was estimated to be 3.2 Mb (3,198,798 bp), which differs from the chromosome sizes of *L. salivarius* (~2.1 Mb) and *L. acidophilus* (~2.0 Mb) but is similar to that of *L. casei* (~3.0 Mb). The estimated GC content of the PS128 draft genome is 44.42%. We obtained 3,103 genes (2,966 protein-coding genes) from NCBI PGAP and 3,202 genes (3,117 protein-coding genes) from RAST annotation service, respectively. We were able to assign 2,298 protein-coding genes (77.48%) according to COG functional categories, and 1,239 protein-coding genes (39.75%) to SEED subsystems (Fig. 1). Among the identified COG categories, the most abundant were transcription (224 genes), carbohydrate transport and metabolism (222 genes), general function prediction only (217 genes), and amino acid transport and metabolism (215 genes). Among the SEED subsystems recognized, the three most abundant were carbohydrates (237 genes), amino acids and derivatives (167 genes), and protein metabolism (132 genes). In searching of genomic sequence of PS128, there was no virulence gene found against virulence factors database (VFDB) [20]; two potential antibiotic genes were found when searching Antibiotic Resistance Genes Database (ARDB) [21], against elfamycin and trimethoprim respectively (Additional file 3).

#### 16S rDNA phylogenetic analysis of PS128 and other sequenced *Lactobacilli*

**Fig. 2:**
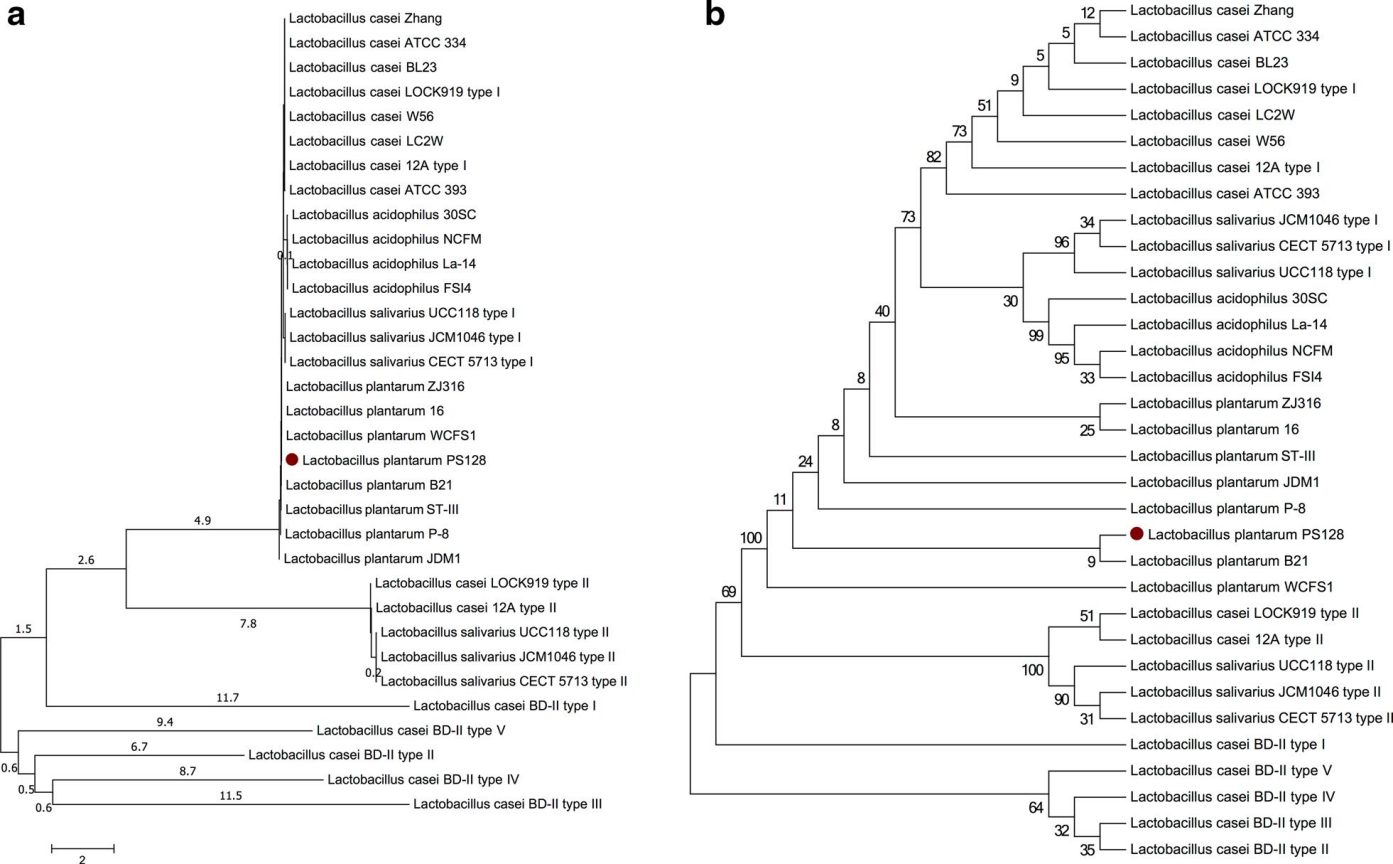
**a** Phylogenetic tree (Neighbor-joining tree) based on 33 16S gene sequences from 24 *Lactobacillus* genomes, drawn to scale, with branch lengths representing evolutionary distances. **b** Bootstrap consensus cladogram drawn so as to represent the percentage of replicates in 1,000 bootstrap tests. Branch lengths are NOT scaled to evolutionary distances. In both panels, PS128 is indicated by a *red dot.*

As indicated by the Neighbor-Joining phylogenetic analysis, the 16S rDNA sequence of strain PS128 is closest to that of strain WCFS1 (which was the first *L. plantarum* strain to have its complete genome published) and that of *L. plantarum* B21 (Fig. 2). PS128, WCFS1, and B21 formed a distinct clade on the tree, while all the strains of *L. plantarum* and *L. acidophilus* were clustered closely together. However, the 16S genes of *L. casei* and *L. salivarius* strains were clustered into different subtrees. Some strains have more than one type of 16S rDNA sequences. For example, *L. casei* BD-II has five 16S rDNAs with five distinct sequence patterns according to multiple sequence alignment. In contrast, *L. plantarum* has a relatively conserved sequence pattern of 16S rDNAs compared to the patterns of *L. casei* and *L. salivarius*. Notably, every strain of *L. plantarum*, including PS128, has only one invariable sequence of 16S rDNA.

#### Comparison of TA-related genes of PS128 with those of other sequenced *L. plantarum* strains

Because *L. plantarum* species have been shown to regulate the immune systems of their hosts and because d-alanylation of TAs has been shown to abolish both pro- and anti-inflammatory immune responses [9], TA-related genes in *L. plantarum* may play key roles in regulating host immune systems. To compare shared and strain-specific TA-related genes, we have summarized the TA-related genes found in published *L. plantarum* genomes and in PS128 in Table 1.

**Table 1:** Comparison of TA-related genes in sequenced *L. plantarum* strains, including PS128

	Counts of TA-related genes in subsystems by strain							
	PS128	16	B21	JDM1	P-8	ST-III	WCFS1	ZJ316
LTA synthase								
*ltaS*	1	1	1	1	1	1	1	1
TA transporter subunits								
*tagG*	1	1	1	1	1	1	1	1
*tagH*	1	1	1	1	1	1	1	1
TA glycosylation proteins								
*gtcA1*	1	1	1	1	1	1	1	1
*gtcA2*	1	1	1	1	1	1	1	1
*gtcA3*	1	1	1	1	1	1	1	1
TA d-alanylation								
*dltX*	1	1	1	1	1	1	1	1
TA synthesis proteins								
*tagF1* (P-8)	1	1	1	0	1	0	1	1
*tagF2* (P-8)	1	1	1	0	1	0	1	1
*tagB* (ST-III)	0	0	0	1	0	1	0	0
*tagB* (ZJ316)	0	0	0	0	0	1	0	1
LTA exporters								
*MPE1*	1	1	1	1	1	1	1	1
*MPE2*	1	0	1	1	0	1	1	1
*MPE3*	1	0	1	0	0	0	0	0
*MPE4*	1	0	1	1	0	0	0	0

*tagF1* and *tagF2* annotations were sourced from P-8; *tagB* covered two different sequence patterns obtained from annotations of *L. plantarum* ST-III and ZJ316.

We found that all the sequenced *L. plantarum* strains, including PS128, shared the same counts of TA-related genes in subsystems that encode a lipoteichoic acid (LTA) synthase (*ltaS*), 2 subunits of the TA transporter [permease protein and ATP-binding protein (*tagG* and *tagH*, respectively)], 3 TA glycosylation proteins (*gtcA1*, *gtcA2*, and *gtcA3*), and a d-alanylation protein (*dltX*). Strains JDM1 and ST-III do not contain genes encoding TA synthesis proteins TagF1 and TagF2, but all the other compared strains do. Only strains JDM1, ST-III, and ZJ316 have TA synthesis protein B. The proteins shared by all of the sequenced *L. plantarum* strains are important for TA metabolism, transport, and modification. Of the genes for the four types of exporters for O-antigen, teichoic acid lipoteichoic acids, the gene for the type I exporter (*MPE1*) is present in all the strains; six strains (PS128, B21, JDM1, ST-III, WCFS1, and ZJ316) have the gene for the type II exporter (*MPE2*), two strains (PS128 and B21) have the gene for the type III exporter (*MPE3*), and three strains (PS128, B21, and JDM1) have the gene for the type IV exporter (*MPE4*).

Comparison of the TA-related genes of PS128 and WCFS1 reveals that the two strains have the same number of genes for LTA synthesis, TA transport, TA glycosylation, TA d-alanylation, and TA synthesis. PS128 has more LTA exporters than WCFS1, although the two strains have similar 16S rDNA sequences. Interestingly, among the eight strains in the comparison, only PS128 and B21 contain four types of LTA exporters. In fact, by comparing the TA-related genes in all of the sequenced *L. plantarum* strains, we found that PS128 is the only strain that has the same distribution pattern of TA-related genes as B21. It is interesting that the distribution pattern in PS128 is the same as that in B21, a *L. plantarum* strain recently reported to have broad-spectrum antimicrobial activity [22]. The abundance of LTA transporter genes in both strains might contribute to immunomodulatory benefits, and strain B21 may also turn out to have anti-inflammatory activity.

### Future directions

The draft genome sequence of *L. plantarum* PS128 may provide substantial genomic information for comparing genetic determinants of interest among *L. plantarum* strains. Our findings raise questions about the degree to which TA-related gene products produced by *L. plantarum* contribute to the bacterial anti-inflammatory activity, and this topic remains to be studied.

### Availability of supporting data

The obtained genome sequence of *L. plantarum* PS128 was deposited in the DDBJ/EMBL/GenBank under accession no. LBHS00000000. The version described in this paper is the first version, LBHS01000000.

## Additional files

**Additional file 1: Figure S1**. PS128 reduced inflammatory response.
**Additional file 2: Figure S2**. Interactions of gene products of TAs-related genes listed in Table 1.
**Additional file 3: Figure S3**. ARDB search result.
