# Supplementary material for: Genome architecture of Lactobacillus plantarum PS128, a probiotic strain with potential immunomodulatory activity
Source: Gut Pathog. 2015 Aug 15;7:22. doi: 10.1186/s13099-015-0068-y (PMC4536865; doi:10.1186/s13099-015-0068-y)
Supplement: Supplementary file 1 — Additional file 1: Figure S1. PS128 reduced inflammatory response. [file 13099_2015_68_MOESM1_ESM.docx]

**Genome architecture of *Lactobacillus plantarum* PS128, a probiotic strain with potential immunomodulatory activity**

Wei-Hsien Liu, Chih-Hsien Yang, Ching-Ting Lin, Shiao-Wen Li, Wei-Shen Cheng, Yi-Ping Jiang, Chien-Chen Wu, Chuan-Hsiung Chang, Ying-Chieh Tsai

**Supplementary figures**


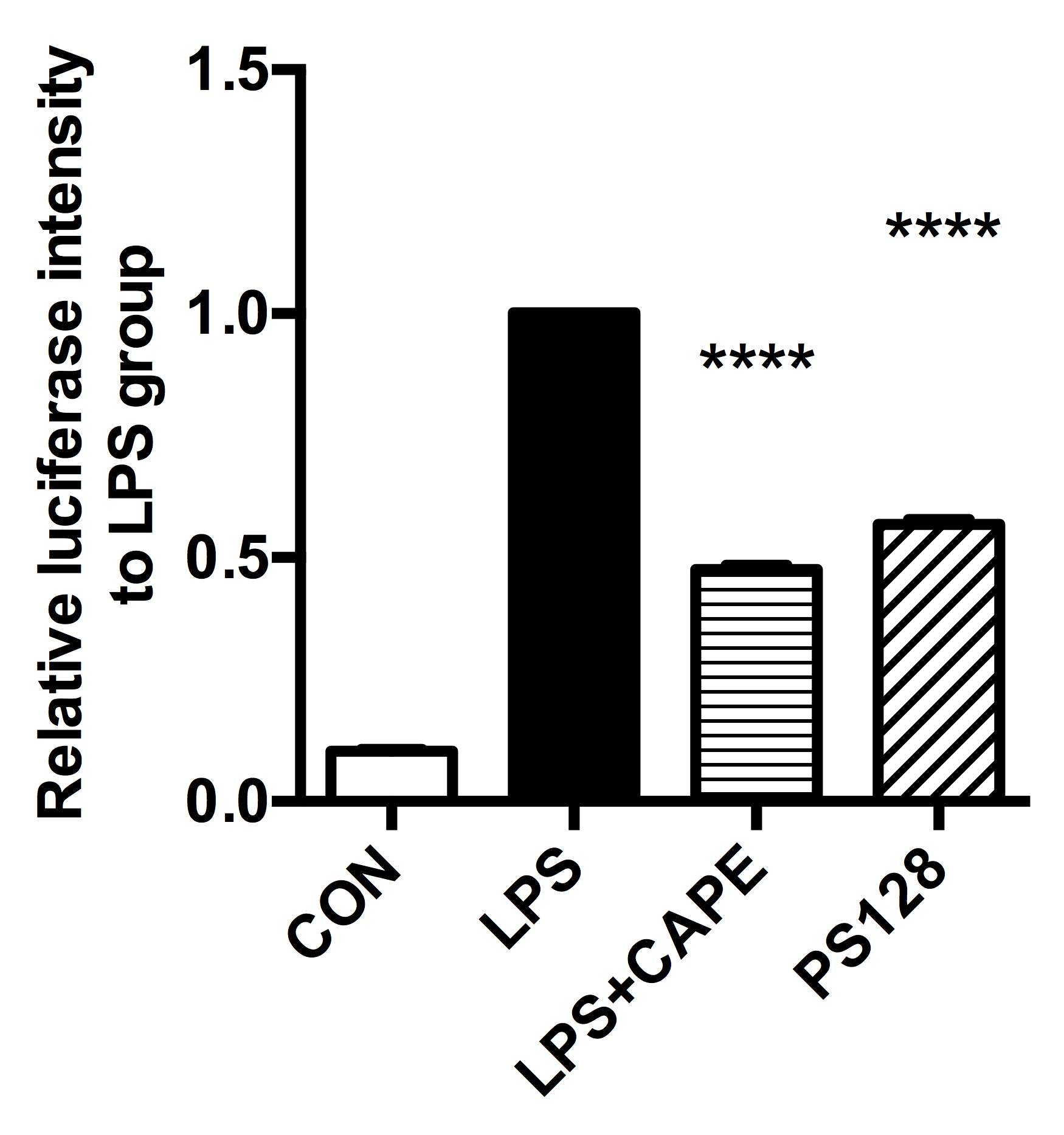


**Captions: PS128 reduced inflammatory response.**

**Figure S1. PS128 reduced inflammatory response.** We assessed the anti-inflammatory effect of PS128 by using RAW 264.7 mouse macrophage cells bearing a luciferase reporter. As shown in the figure, the inducible nitric oxide synthase (iNOS) production was increased upon lipopolysaccharide (LPS, 1 μg/mL) treatment. On the other hand, iNOS was reduced when the cells were treated with caffeic acid phenethyl ester (CAPE), which is known to reduce pro-inflammatory cytokine production [1]. Similarly, addition of heat-killed PS128 at a final does of 10^7^ CFU/mL reduced iNOS production to the level near the LPS+CAPE group. Data were expressed as mean ± SD. Statistical difference was calculated by one-way ANOVA with Tukey's post-test. *****p* < 0.0001 compared to the LPS group.

**Reference**

1. Zhao WX, Wang L, Yang JL, Li LZ, Xu WM, Li T. Caffeic acid phenethyl ester attenuates pro-inflammatory and fibrogenic phenotypes of LPS-stimulated hepatic stellate cells through the inhibition of NF-kappaB signaling. Int J Mol Med. 2014;33:687.
