# Supplementary material for: Genome architecture of Lactobacillus plantarum PS128, a probiotic strain with potential immunomodulatory activity
Source: Gut Pathog. 2015 Aug 15;7:22. doi: 10.1186/s13099-015-0068-y (PMC4536865; doi:10.1186/s13099-015-0068-y)
Supplement: Supplementary file 2 — Additional file 2: Figure S2. Interactions of gene products of TAs-related genes listed in Table 1. [file 13099_2015_68_MOESM2_ESM.docx]

**Genome architecture of *Lactobacillus plantarum* PS128, a probiotic strain with potential immunomodulatory activity**

Wei-Hsien Liu, Chih-Hsien Yang, Ching-Ting Lin, Shiao-Wen Li, Wei-Shen Cheng, Yi-Ping Jiang, Chien-Chen Wu, Chuan-Hsiung Chang, Ying-Chieh Tsai

**Supplementary figures**

**Caption: Interactions of gene products of TAs-related genes listed in Table 1.**

**Figure S2. Interactions of gene products of TAs-related genes listed in Table 1.** This diagram was based on a previous report showing the type I LTA synthesis machinery and lipid turnover in *Staphylococcus aureus* [1], and a review article showing conserved intracellular WTA biosynthesis steps in *Bacillus sbutilis* and *S. aureus* [2]. This diagram only shows our current understanding based on previous studies and gene annotations of *L. plantarum* genomes. The TA-related processes in the *L. plantarum* still require further studies. MPE: LTA exporter; LtaS: LTA synthase; Gtc: TA glycosylation proteins; DltX: TA D-alanylation protein; TagG and TagH: TA transporter subunits; TagF and TagB: TA synthesis protein.

**Reference**

1. Percy MG, Grundling A. Lipoteichoic acid synthesis and function in gram-positive bacteria. Annu Rev Microbiol. 2014;68:81.

2. Brown S, Santa Maria JP, Jr., Walker S. Wall teichoic acids of gram-positive bacteria. Annu Rev Microbiol. 2013;67:313.
